# The role of the AMOP domain in MUC4/Y-promoted tumour angiogenesis and metastasis in pancreatic cancer

**DOI:** 10.1186/s13046-016-0369-0

**Published:** 2016-06-10

**Authors:** Jie Tang, Yi Zhu, Kunling Xie, Xiaoyu Zhang, Xiaofei Zhi, Weizhi Wang, Zheng Li, Qun Zhang, Linjun Wang, Jiwei Wang, Zekuan Xu

**Affiliations:** Department of General Surgery, the First Affiliated Hospital of Nanjing Medical University, Nanjing, Jiangsu China; Department of Pediatric Surgery, Nanjing Children’s Hospital Affiliated to Nanjing Medical University, Nanjing, Jiangsu China; Department of General Surgery, the People’s Hospital of Bozhou, Bozhou, Anhui China; Department of General Surgery, Huai’an People’s Hospital, Xuzhou Medical College, Huai’an, Jiangsu China; Department of General Surgery, Affiliated Hospital of Nantong University, Nantong, Jiangsu China

**Keywords:** MUC4/Y-AMOP domain, Tumour angiogenesis, Metastasis, NOTCH3, Pancreatic cancer

## Abstract

**Background:**

MUC4 is a high molecular weight membrane protein that is overexpressed in pancreatic cancer (PC) and is associated with the development and progression of this disease. However, the exact mechanisms through which MUC4 domains promote these biological processes have rarely been studied, partly because of its high molecular weight, difficulty to overexpress it. Here, we use MUC4/Y, one of the MUC4 transcript variants, as a model molecule to investigate the AMOP-domain of MUC4(MUC/Y).

**Methods:**

We used cell proliferation, migration, invasion and tube formation assays in vitro to explore the abilities of AMOP domain in PC. In vivo, the matrigel plug assay, orthotopic implantation and Kaplan-Meier survival curves were used to check the results we observed in vitro. Finally, we discovered the underlying mechanism through western blot and immunofluorescence.

**Results:**

We found that MUC4/Y overexpression could enhance the angiogenic and metastatic properties of PC cells, both in vitro and in vivo. However, the deletion of AMOP domain could cutback these phenomena. Additionally, Kaplan-Meier survival curves showed that mice injected with MUC4/Y overexpressed cells had shorter survival time, compared with empty-vector-transfected cells (MUC4/Y-EV), or cells expressing MUC4/Y without the AMOP domain (MUC4/Y-AMOP^△^). Our data also showed that overexpression of MUC4/Y could activate NOTCH3 signaling, increasing the expression of downstream genes: VEGF-A, MMP-9 and ANG-2.

**Conclusions:**

The AMOP domain had an important role in MUC4/Y (MUC4)-mediated tumour angiogenesis and metastasis of PC cells; and the NOTCH3 signaling was involved. These findings provided new insights into PC therapies. Our study also supplies a new method to study other high molecular membrane proteins.

**Electronic supplementary material:**

The online version of this article (doi:10.1186/s13046-016-0369-0) contains supplementary material, which is available to authorized users.

## Background

Pancreatic Cancer (PC) is the fourth leading cause of death due to cancer worldwide [1]. The incidence and number of deaths caused by PC have been gradually increasing, even as the incidence and mortality of other common cancers have been declining. Surgical resection remains the only chance for cure, but approximately 80–85 % of patients present with advanced un-resectable state at the time of diagnosis. Unfortunately, PC responds poorly to most chemotherapeutic agents; thus, the 5 year survival rate is only approximately 4 % [2]. Hence, the biological mechanisms that contribute to the development and progression of PC need to be investigated.

MUC4, a large membrane-anchored glycoprotein, is aberrantly expressed in various types of cancers and inflammatory diseases. Its expression is undetectable in normal pancreatic tissue and chronic pancreatitis, but it is highly expressed in pancreatic intraepithelial neoplasia and PC [3]. We and other researchers have reported that MUC4 is involved in various biological properties of PC cells, including growth, apoptosis, invasion, tumour angiogenesis and drug resistance [4–13].

Alternative splicing, which is often dysregulated in cancer, can produce various isoforms of genes with differential properties and therefore diverse effects on cancer progression [14, 15]. For the MUC4 gene, 24 distinct splice transcripts have been isolated from various tissue samples as well as cell lines, named from sv0 (the full-length MUC4) to sv21-MUC4, MUC4/X, and MUC4/Y [16]. Splice variants of MUC4 are present in pancreatic intraepithelial neoplasia and PC but not in the normal pancreas or in chronic pancreatitis [17–21]. Thus, exploring the function of MUC4 splice variants at the protein level may help us to determine their potential functions in pancreatic carcinomas.

MUC4/Y, a MUC4 splice variant, lacks the fragment translated from exon 2 (encodes the tandem repeat domain), including the NIDO, AMOP, vWD, and EGF-like domains, the trans-membrane domain (TM), and the cytoplasmic tail [15, 16]. MUC4/Y is named by analogy with MUC1/Y. Compared with MUC1, MUC1/Y lacks the randomly repeated amino acids. As a well-established transcript form of MUC1, MUC1/Y has important functions in tumour initiation and progression [22–24]. We have reported that MUC4/Y could stimulate PC cell line proliferation, invasion and suppress apoptosis [25, 26]. The role of these domains of MUC4/Y is of interest.

The AMOP domain is a novel extracellular domain that is found in some splice variants of MUC4. It is uncommon in the genome, and it has been described in only 4 proteins so far: MUC4, SUSD2, ISM1, and ISM2. The exact features of AMOP domain remain unknown, although it has been suggested functional in cell adhesion [27–29]. In this study, we investigated whether the deletion of the AMOP domain could alter MUC4(MUC4/Y)-mediated tumour biological processes in PC cells. We found that the deletion of AMOP domain could reverse the tumour angiogenesis and metastasis induced by MUC4/Y. The underlying mechanism was the activation of NOTCH3 downstream genes (VEGF-A, MMP-9 and ANG-2) by AMOP domain. This mechanism could be a potential therapeutic target of PC.

## Methods

### Cell culture

The human pancreatic cancer cell lines PANC-1 and MiaPaCa-2 were purchased from the Shanghai Institutes for Biological Sciences, Chinese Academy of Sciences. They were maintained in DMEM containing 10 % foetal bovine serum (FBS) (Wisent, Canada) and 1 % penicillin/streptomycin (HyClone, Thermo, USA). Human umbilical venous endothelial cells (HUVECs) (ATCC, USA) were cultured in Endothelial Cell Growth Medium. All cell lines were grown in a humidified chamber supplemented with 5 % CO2 at 37 °C.

### Lentiviral production and Infection

The MUC4/Y (NM_004532.4, 167736352) and MUC4/Y-AMOP^Δ^ were synthesised artificially and cloned into the pUC57 plasmid (Genscript Co., China). Lentiviral production was achieved using the pUC57 plasmid carrying MUC4/Y (Shanghai SBO Medical Biotechnology Co., China), with a three-plasmid system of pCDH-CMV-MCS-EF1-Puro, pCD/NL-BH*DDD and pLTR-G. The pancreatic cancer cell lines PANC-1 and MiaPaCa-2 were infected following the manufacturer’s instructions. These cell lines were selected by 2 μg/ml bulk puromycin-resistant culturing (Sigma, USA) for one week. Then, MUC4/Y and MUC4/Y-AMOP^Δ^ expression levels were analysed by real time qPCR and western blotting assays. The cells were then subjected to additional assessments as follows.

### mRNA extraction and real-time qPCR

Total RNA was extracted from cell lines using TRIzol reagent (Life Technologies, USA), following the manufacturer’s protocols. After spectrophotometric quantification, 1 μg of total RNA was used for reverse transcription (RT) in a final volume of 20 μl with the Prime-Script RT Reagent (Takara, Japan), according to the manufacturer’s instructions. The amounts of cDNA used for the amplification of the target genes were normalised to the human GAPDH gene. Real-time qPCR was performed on a Step One Plus Real-Time PCR System (Life Technologies, USA) using Fast Start Universal SYBR Green Master Mix (Roche, USA). The primers were as follows: MUC4/Y, forward: 5′-GTCCCAGGAATGACAACAC-3′, reverse: 5′-AATGGTGGAAATGATGGTCTG-3′; GAPDH, forward:5′-ATCTCTGCCCCCTCTGCTGA-3′, reverse: 5′-GATGACCTTGCCCACAGCCT-3′; NOTCH1, forward: 5′-GAGGCGTGGCAGACTATGC-3′, reverse: 5′-CTTGTACTCCGTCAGCGTGA-3′; NOTCH2, forward: 5′-CAACCGCAATGGAGGCTATG-3”; reverse: 5′-GCGAAGGCACAATCATCAATGTT-3′; NOTCH3, forward: 5′-TGGCGACCTCACTTACGACT-3′, reverse: 5′-CACTGGCAGTTATAGGTGTTGAC-3′; NOTCH4, forward: 5′-GATGGGCTGGACACCTACAC-3′, reverse: 5′-CACACGCAGTGAAAGCTACCA-3′; ANG-2, forward: 5′-CTGGGCGTTTTGTTGTTGGTC-3′, reverse: 5′-GGTTTGGCATCATAGTGCTGG-3′. Hes-1, forward: 5′-TGGATGCTCTGAAGAAAGATAGC-3′; reverse: 5′-CTCGGTACTTCCCCAGCAC-3′. The 2^-ΔΔCT^ method was used to calculate relative expression levels. Each real-time PCR was performed in triplicates and independently repeated for three times.

### Western blotting

Protein from the cell extracts was resolved by electrophoresis and transferred to poly vinylidene difluoride membrane (PVDF). After blocking with 5 % non-fat milk in Tris-buffered saline, membranes were incubated with specific antibodies at 4 °C overnight. The membrane was then incubated with horseradish peroxidase labeled secondary antibodies. Proteins were visualized with the Super Signal West Femto Maximum sensitivity substrate kit (Thermo Scientific, Logan, UT). Western blots were quantified using the software Image Pro Plus version 6. The antibodies to ANG-2 (SC-7015) and GAPDH (SC-365062) were purchased from Santa Cruz Biotechnology (USA). The antibodies to MUC4/Y (MUC4) (#ab60720), VEGF-A (#ab51745), CD31 (#ab28364) and Hes-1 (#ab71559) were bought from Abcam (USA). The selected monoclonal antibody (#ab60720, Abcam, UK) is specifically directed against amino acids 79–189 of human MUC4, which are included in the protein expressed by the MUC4/Y target gene, including the AMOP, NIDO, VWD, and the EGF-like domains. The antibodies to NOTCH3 (#5276), and MMP-9 (#13667) were from Cell Signalling Technology (USA). Pre-stained markers (Thermo Scientific, USA) were used as internal molecular weight standards. Each blot was independently repeated three times.

### In vitro HUVEC tube -formation assay

PANC-1 cells were cultured as described above. When the cells reached 80 % confluence, the culture medium was changed to DMEM without FBS. Following an additional 24 h of culturing, the supernatant was collected as conditioned medium and stored at −80 °C. After thawing at 4 °C overnight, the 50 μl Matrigel (BD, USA) was coated in a 96-well plate and then incubated at 37 °C for at least 1 h to gel. HUVECs were suspended at a density of 2 × 10^5^ cells/ml in the different supernatants. The cell suspensions (100 μl) were added to each Matrigel-coated well. DMEM was used as the negative control. After 5–12 h, tube images were taken using a digital camera attached to an inverted phase-contrast microscope. The total tube length in each well was measured and calculated using Image-Pro Plus (IPP) software.

### HUVEC proliferation assay

HUVECs were suspended at a density of 2 × 10^4^ cells/ml, and 100 μl cell suspensions were seeded into 96-well plates. After 24 h, the medium was changed to conditioned medium, as described above. Cell proliferation was assessed using the Cell Counting Kit-8 (CCK8) (Dojindo Laboratories, Japan), following the manufacturer’s protocol. The cells were stained at the indicated time point with 10 μl CCK8 for 4 h at 37 °C in a CO_2_incubator. The absorbance at 450 nm (OD value) was measured using a micro-plate reader, and the absorbance at 630 nm was used as a reference. The average OD values were used to represent the total cell numbers of each group. All tests were performed in triplicate.

### HUVEC migration and invasion assays

Cell migration and invasion assays were performed using a chamber 6.5 mm in diameter with an 8 μm pore size (Corning, USA). The upper chambers were seeded with 1 × 10^4^ HUVEC cells. Subsequently, the different conditioned media were added to the lower chamber. For the invasion assay, the top chamber was coated with 100 μl of 1 mg/ml Matrigel (BD, USA). The cells were incubated at 37 °C for 36 h. After incubation, the cells that did not migrate through the pores and remained in the upper chamber were removed by scraping the membrane with a cotton swab. The migrated cells on the lower side of the membrane were stained with 0.1 % crystal violet (Sigma, USA) for 20 min at 37 °C, followed by washing with PBS and photographing in 10 random fields of view at 10× magnification. The cell numbers were counted and expressed as the average number of cells/field of view. Three independent experiments were performed in each case.

### PANC-1 migration and invasion assays

Cell migration and invasion assays were performed using a chamber 6.5 mm in diameter with an 8 μm pore size (Corning, USA). For migration assays, 5 × 10^4^ PANC-EV, PANC-MUC4/Y and PANC-MUC4/Y-AMOP^Δ^ cells were suspended separately in serum-free DMEM plated in the top chamber of inserts. Then, 0.5 ml of 10 % serum-containing DMEM was added to the lower chamber of the well and the cells were allowed to migrate under chemotactic drive at 37 °C for 24 h; the cells in the upper chamber were then removed using cotton swabs. For invasion assays, cells (5 × 10^4^) were seeded on Matrigel-coated membrane inserts. Cells migrating or invading into the bottom of the membrane were stained with 0.1 % crystal violet for 20 min at 37 °C, followed by washing with PBS. Ten random fields from each membrane were photographed and counted for statistical analysis.

### Animals

Four-week-old male nude mice (BALB/c-nu (nu/nu)) were purchased from the Shanghai Experiment Animal Center (Chinese Academy of Sciences, China) and housed in specific pathogen-free conditions. This study was conducted in strict accordance with the recommendations in the Guide for the Care and Use of Laboratory Animals of the Ministry of Health, China. The Ethics Committee of the First Affiliated Hospital of Nanjing Medical University (Permit Number: 2012-SRFA-093) approved the protocol.

### Matrigel plug assay

We conducted a Matrigel plug assay to investigate the tumour angiogenesis properties of MUC4/Y and domain deletion. Twenty-four male mice were randomly divided into three groups. PANC-EV, PANC-MUC4/Y, and PANC-MUC4/Y-AMOP^Δ^ cells were re-suspended at 2 × 10^7^ cells/ml in serum-free medium. Aliquots of cells (0.4 ml, 8 × 10^6^ cells) were mixed with 0.4 ml Matrigel and unilaterally injected into the flank of each mouse (100 μl mixture/per flank). Matrigel mixed with medium served as a negative control. The Matrigel plugs were removed 15 days after implantation. Half of the plugs were used for the measurement of haemoglobin content using Drabkin’s reagent (Sigma, USA). The remaining part of the plugs were fixed in 4 % formalin, embedded in paraffin and used for IHC analysis.

### In vivo tumour metastasis

To investigate the functional consequences of the MUC4/Y and MUC4/Y-AMOP domain on the metastatic properties of PC cells, orthotopic implantation was carried out. Twenty-four mice were randomly divided into three groups (PANC-EV, PANC-MUC4/Y, PANC-MUC4/Y-AMOP^Δ^). The PANC-1 derived cells were harvested from sub-confluent cultures and re-suspended in PBS at a concentration of 2 × 10^6^ cells/100 μl. Single-cell suspensions of >95 % viability were used for the assays. The animals were anesthetised with intra-peritoneal 0.1 % pentobarbital sodium. All surgical procedures were performed under sterile conditions. Two million cells suspended in 100 μl of PBS were injected into the pancreas by a 30-gauge needle. The animals were monitored every two days. To determine tumour metastasis, mice were euthanised by CO_2_ asphyxiation and autopsied 45 days after the implantation of the tumour cells. Regional and distant lymph nodes, liver, lung and other organs suspected of harbouring metastases were routinely formalin-fixed, embedded, sectioned, and stained with hematoxylin and eosin using standard techniques for microscopic examination.

### Immunohistochemistry (IHC)

All specimens were fixed in 4 % formalin and embedded in paraffin before IHC analysis. All procedures with reference to our previous reports [15]. The tumour slides were examined in a blinded manner. Five fields were selected for examination, and the percentage of positive tumour cells and cell-staining intensity were determined.

Micro-vessel density (MVD) was counted by CD31 staining. Five areas with the highest MVD were chosen for counting under 200× magnification. The average number of vessels in the five areas was considered as the MVD level of the case. Any brown-staining endothelial cells or endothelial cell clusters that were clearly separate from adjacent micro-vessels, tumour cells, and other connective tissue elements, were recorded as a single countable micro-vessel. Even those distinct clusters of the stained endothelial cells that might come from the same vessel snaking its way in and out of the section were considered as separate micro-vessels.

### VEGF-A assay

Because VEGFA and MMP9 are secreted factors, they mainly work through paracrine. So we used the commercial kits to test the activity of VEGFA and MMP9 in the conditioned medium of different groups. The PANC-EV, PANC-MUC4/Y or PANC-MUC4/Y-AMOP^Δ^ cells were seeded in six-well plates (1.5 × 10^5^ per well), and incubated at 37 °C. After 24 h, the cell culture supernatant was harvested and cell counts were performed after trypsinisation. After collection, the medium was spun at 800 × *g* for 3 min at 4 °C to remove cell debris. The supernatant was either frozen at −80 °C for later activity assays or assayed immediately using commercially available ELISA kits (R&D systems, USA).

### MMP-9 activity assay

The PANC-EV, PANC-MUC4/Y or PANC-MUC4/Y-AMOP^Δ^ cells were seeded in six-well plates and incubated at 37 °C. After 24 h, the medium was removed and the cells were washed with serum-free medium. The cells were then incubated in serum-free medium for 24 h. MMP-9 activity in the medium was detected using the Fluorokine E Human MMP-9 Activity Assay kit (R&D systems, USA), according to the manufacturer’s protocol.

### Statistical analysis

Statistical analysis was performed using the SPSS (Statistical Package for the Social Sciences) software package (Version 18.0). Quantitative data are presented as the mean ± SD. Differences in the mean values of two samples were analysed by Student’s *t*-test. The Kaplan-Meier method was used to determine the survival distributions and overall survival rates, and the significance of differences between survival rates was calculated by the log-rank test. *P* < 0.05 was considered significant.

## Results

### Construction of MUC4/Y and MUC4/Y-AMOP^Δ^ overexpression cell lines

**Fig. 1:**
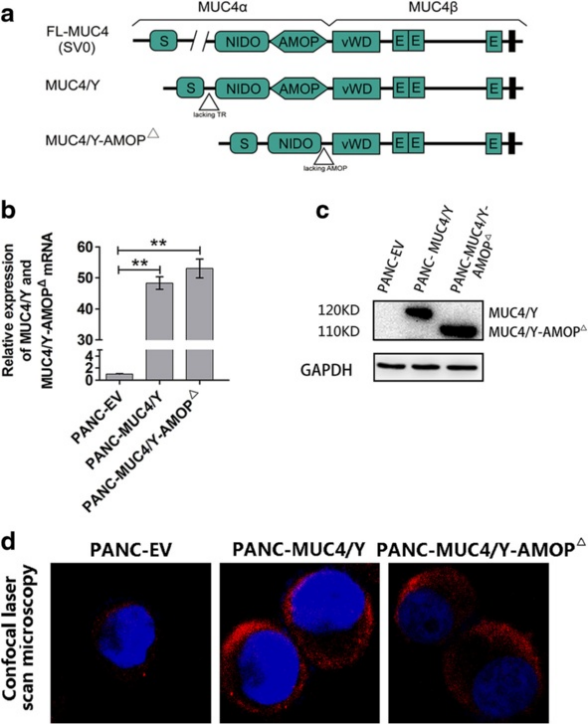
MUC4/Y and MUC4/Y-AMOP domain-deletion constructs and their stable expression in PANC-1 cells. **a** The structure diagram of the MUC4, MUC4/Y and MUC4/Y-AMOP^△^. Different domains present in MUC4 protein are the central large tandem repeat domain, NIDO, AMOP, vWD, and 3 carboxyl-terminal located EGF domains. **b** MUC4/Y expression was analysed by real-time PCR in different PANC-1 derived sub-lines. GAPDH was used as an internal control. **c** Western blotting was conducted to analyse the protein level of MUC4/Y in different groups. GAPDH was probed as an internal control. **d** Cell confocal immunofluorescence assay showed that the MUC4/Y and the truncated protein (MUC4/Y-AMOP^Δ^) could anchor on the cytomembrane and cytoplasm.**P < 0.01

To investigate the possible role of the MUC4/Y-AMOP domain, we constructed MUC4/Y and MUC4/Y-AMOP^Δ^ lentivirus vectors and infected the PANC-1 cells. The structure organization of MUC4, the MUC4/Y, and the truncated protein (MUC4/Y-AMOP^Δ^), were shown in Fig. 1a. The exact “aa” position for the AMOP domain that was deleted was shown in Additional file 1: Table S1. PANC-1 cells without MUC4/Y (MUC4) expression, as we previously reported [16], were chosen to establish the stable cells lines. Real-time PCR and western blotting assays were used to confirm the expression of MUC4/Y (PANC-MUC4/Y) or the MUC4/Y without the AMOP domain (PANC-MUC4/Y-AMOP^Δ^) at mRNA (Fig. 1b) and protein levels (Fig. 1b). The MUC4/Y-AMOP^△^ protein (~110 KD) was smaller than MUC4/Y(~120KD), perhaps because of the AMOP domain deletion (Fig. 1c). Cell immunofluorescence assay showed that the MUC4/Y and the truncated protein (MUC4/Y-AMOP^Δ^) could anchor on the cyto-membrane and cytoplasm. It clearly pointed out that the deletion of the AMOP domain would not change the localization of the MUC4/Y protein (Fig. 1d).

For the reproducibility of the results, we chose one more PC cell line (MiaPaCa-2, MIA) to repeat the above experiments. We found the similar phenomenon as we observed in PANC-1 cell line (Additional file 1: Figure S1a, b and c).

### MUC4/Y-AMOP domain regulates the processes of tumour angiogenesis in vitro

To confirm the role of the MUC4/Y-AMOP domain in tumour angiogenesis, the HUVEC cells tube formation, migration, invasion and proliferation assays were performed in vitro*.*

**Fig. 2:**
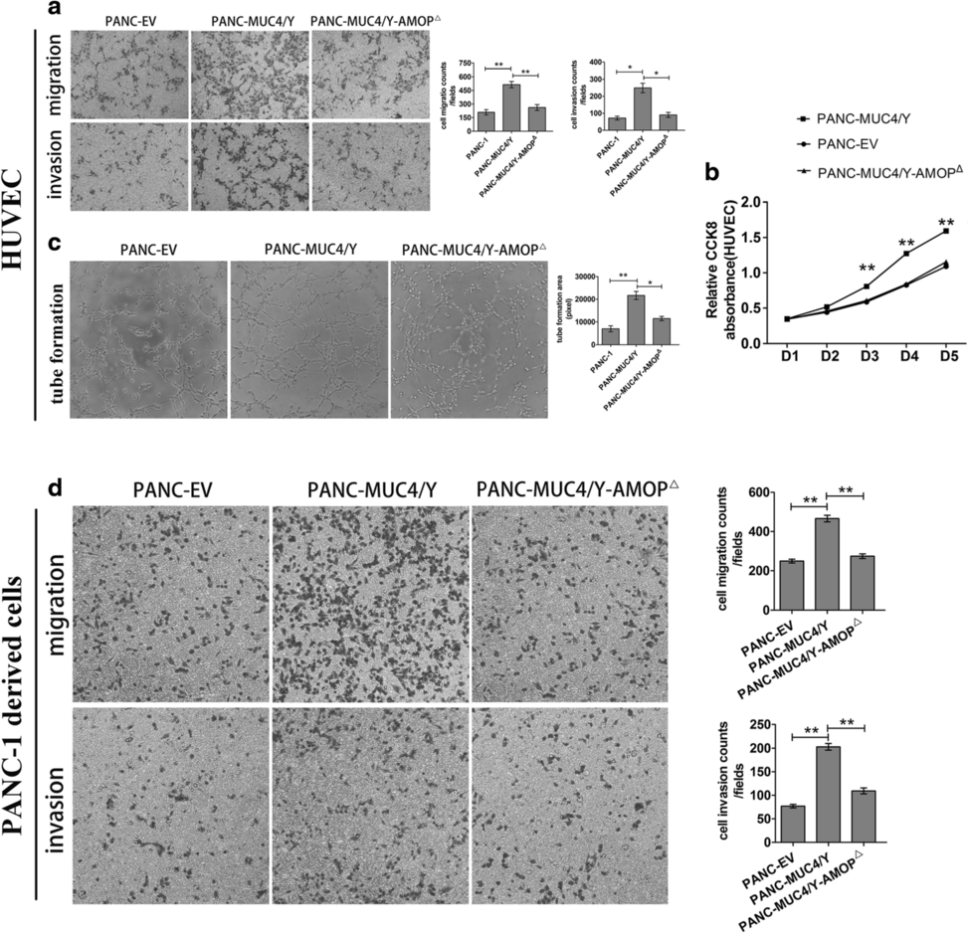
Effects of the MUC4/Y-AMOP domain-deletion on the migration and invasion of PANC-1 derived sub-lines and the conditioned medium on the tube formation, proliferation, migration, and invasion of HUVEC. **a** Trans-well migration and Matrigel invasion assays of HUVEC were performed in the conditioned medium of different groups. **b** CCK8 assay. The OD value was used to assess the proliferation of HUVEC in the conditioned medium of different groups. **c** Tube formation assays of HUVEC. The area of network was calculated with Image Pro Plus 6.0. **d** Trans-well migration and Matrigel invasion assays of PANC-1 derived sub-lines. The number of cells that migrate or invade to the lower surface of the inserts chamber were stained and photographed. *P < 0.05, **P < 0.01

We used cell migration and matrigel invasion assays to investigate the effects of the MUC4/Y-AMOP domain in HUVEC migration and invasion. The number of HUVEC that migrated to the lower surface of the insert chamber was increased by the conditioned medium from MUC4/Y group cells compared with MUC4/Y-AMOP^Δ^ and control (EV) groups both in cell migration and matrigel invasion assays (Fig. 2a, Additional file 1: Figure S2A).

We also used the CCK8 assay to assess the effects of HUVEC proliferation in the conditioned medium of different groups. The proliferation of HUVEC in the conditioned medium of the MUC4/Y overexpression group was increased compared with the other two groups (Fig. 2b, Additional file 1: Figure S2B).

Next, tube-formation assays with HUVEC were performed using different conditioned medium (EV, MUC4/Y, and MUC4/Y-AMOP^Δ^). Consistent with the results above, the deletion of AMOP domain could decrease the tube-forming capacity of HUVEC cells that MUC4/Y enhanced (Fig. 2c, Additional file 1: Figure S2C).

### MUC4/Y-AMOP domain promotes the motility and invasive ability of PC cells

The trans-well assay was used to determine the role of the MUC4/Y-AMOP domain in PC cell migration and invasion. The number of cells that migrate or invade to the lower surface of the insert chamber was greater in the MUC4/Y overexpressed cells than the cells without MUC4 expression or cells with MUC4/Y-AMOP^Δ^(Fig. 2d, Additional file 1: Figure S2D).

### MUC4/Y-AMOP domain facilitates tumour angiogenesis in vivo

**Fig. 3:**
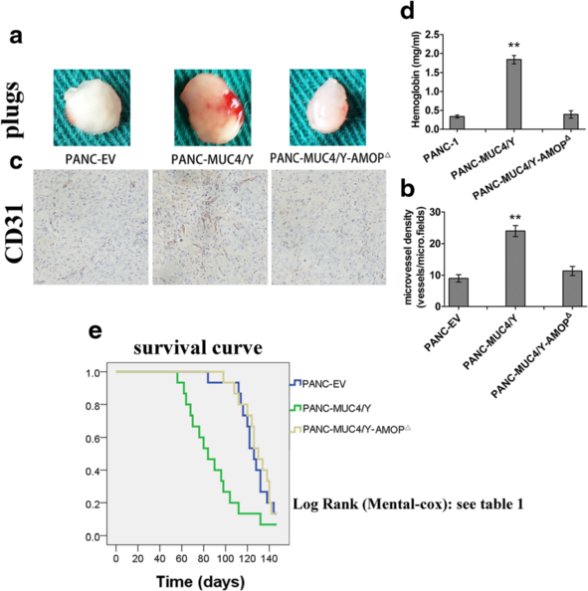
Matrigel plug assay and the correlation between MUC4/Y-AMOP domain and mouse survival. **a** Cells were suspended in Matrigel and DMEM and subcutaneously into the flanks of mice (*n* = 8). After 15 days, the Matrigel plugs were removed and photographed. Representative Matrigel plugs from the PANC-EV, PANC-MUC4/Y, and PANC-MUC4/Y-AMOP^△^groups are shown. **b** The relative amount of angiogenesis was analysed based on the RBC haemoglobin level, determined using the Drabkin method. The relative haemoglobin content is the haemoglobin level (mg) divided by the final volume of each plug. **c** CD31 immunohistochemistry was performed on the Matrigel plugs from the PANC-EV, PANC-MUC4/Y, and PANC-MUC4/Y-AMOP^△^groups. **d** Microvessel density of CD31 immunohistochemistry. Each slide was evaluated with five fields, and the data were analysed as the mean vessel number of these five fields. **e** Kaplan-Meier survival curves in 45 mice with PANC-1 derived sub-lines orthotopically implanted. The p values were calculated by the log-rank test (Table 1)

To further study the effects of the MUC4/Y-AMOP domain on tumour angiogenesis in PC, in vivo experiments were performed using the Matrigel plug assay. PANC-EV, PANC-MUC4/Y and PANC-MUC4-AMOP^Δ^ cells were suspended in a Matrigel/DMEM mixture and subcutaneously injected into mice (Fig. 3a). Consistent with the previous results, the PANC-MUC4/Y cells formed more vessels (MVD) compared with the PANC-MUC4/Y-AMOP^Δ^ and PANC-EV groups (Fig. 3b and c). Furthermore, the haemoglobin concentration was measured following Matrigel dissolution to assess the number of functional vessels. The PANC-MUC4/Y group also showed significantly higher levels of haemoglobin (Fig. 3d).

### MUC4/Y-AMOP domain is an independent predictor of unfavourable outcome of the tumour-bearing nude mice

PANC-1-derived cells were orthotopically injected into the head of the pancreas on day 0 as described above. The mice were randomised into three groups (15 mice/group): control group (PANC-EV), MUC4/Y overexpression group (PANC-MUC4/Y), and MUC4/Y without AMOP-domain overexpression group (PANC-MUC4/Y-AMO^△^). The endpoint of this study was the death of the mice. The efficacy study was terminated on day 147 (21 weeks) post-tumour cell injection. As shown in Fig. 3e, the Kaplan-Meier survival curves showed that mice injected with PANC-MUC4/Y cells had shorter survival time than those injected with PANC-EV or PANC-MUC4/Y-AMOP^Δ^. The control group had a median survival of 130 days, which was much longer than the 76 days in MUC4/Y overexpression group. The median survival time of the PANC-MUC4/Y-AMOP^△^ group was not significantly different compared with the control group (Fig. 3e and Table 1).

**Table 1:** Log Rank (Mantel-Cox)----Pairwise Comparisons

Group	PANC-EV	PANC-MUC4/Y	PANC-MUC4/Y-AMOP^△^
	P value	P value	P value
PANC-EV		0.004^**^	0.695
PANC-MUC4/Y	0.004^**^		0.002^**^
PANC-MUC4/Y-AMOP^△^	0.695	0.002^**^	

The endpoint of the assay is the death of the mice. **p<0.01

### The MUC4/Y-AMOP domain promotes metastasis of PANC-1 cells in vivo

**Fig. 4:**
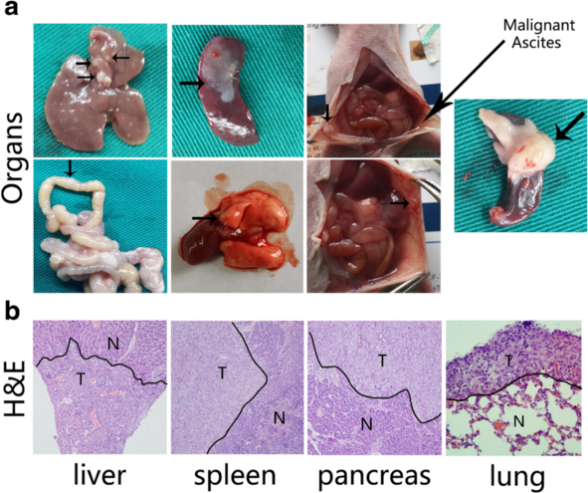
Macroscopic and microscopic examination of organs with metastatic lesions and orthotopic pancreatic tumours. 24 mice were randomly divided into three groups (PANC-EV, PANC-MUC4/Y, PANC-MUC4/Y-AMOP^Δ^). The different groups PANC-1 derived cells (2 × 10^6^ cells/100 μl) were orthotopic implanted into pancreas. Forties five dayed later, mice were euthanised by CO_2_ asphyxiation and autopsied (**a**) The macroscopic examination of organs with metastatic lesions (includes liver, spleen, lung, peritoneum, and mesentery), malignant ascites and orthotopic pancreatic tumours. The arrows pointed the metastatic nodes. **b** H&E staining was used to the histological change. Normal (N) and tumour lesions (T) in different organs are separated as indicated

We confirmed the metastatic effect of the MUC4/Y-AMOP domain in vivo using orthotopic injection with infected cells into nude mice. The overall tumour incidence of pancreas among the three groups (8mice/group, 100 %) has no significant difference. However, there were higher incidences of metastasis at different sites in the MUC4/Y overexpressed group compared with the other two groups, including liver, mesenteric, lung and malignant ascites, suggesting that the AMOP domain had an important role in the metastasis. The incidence of metastasis in spleen was same (8mice, 100 %) in the three groups, indicating that MUC4/Y might have no impact on the local invasion (Table 2). We found no significant difference of metastasis in peritoneum, possibility due to the limited number of sample. We confirmed by visual and histological inspection. The arrows pointed to the metastatic nodes (Fig. 4a and b)

**Table 2:** The incidence rate of the tumour at different sites

Group	Organ						Malignant Ascites (%)
	Pancreas	Liver	Spleen	Peritoneum	Mesentery	Lung	
PANC-MUC4/Y *vs* PANC-EV	8/8 vs 8/8	8/8 vs 1/8	8/8 vs 8/8	4/8 vs 1/8	4/8 vs 0/8	5/8 vs 0/8	4/8 vs 0/8
	*P* > 0.05	*P* < 0.01	*P* > 0.05	*P* > 0.05	*P* < 0.05	*P* < 0.05	*P* < 0.05
PANC-MUC4/Y *vs* PANC-MUC4/Y-AMOP^Δ^	8/8 vs 8/8	8/8 vs 2/8	8/8 vs 8/8	4/8 vs 1/8	4/8 vs 0/8	5/8 vs 0/8	4/8 vs 0/8
	*P* > 0.05	*P* < 0.01	*P* > 0.05	*P* > 0.05	*P* < 0.05	*P* < 0.05	*P* < 0.05
PANC-EV *vs* PANC-MUC4/Y-AMOP^Δ^	8/8 vs 8/8	1/8 vs 2/8	8/8 vs 8/8	1/8 vs 1/8	0/8 vs 0/8	0/8 vs 0/8	0/8 vs 0/8
	*P* > 0.05	*P* > 0.05	*P* > 0.05	*P* > 0.05	*P* > 0.05	*P* > 0.05	*P* > 0.05

### The MUC4/Y-AMOP domain is involved in MUC4/Y up-regulation of the expression of NOTCH3

**Fig. 5:**
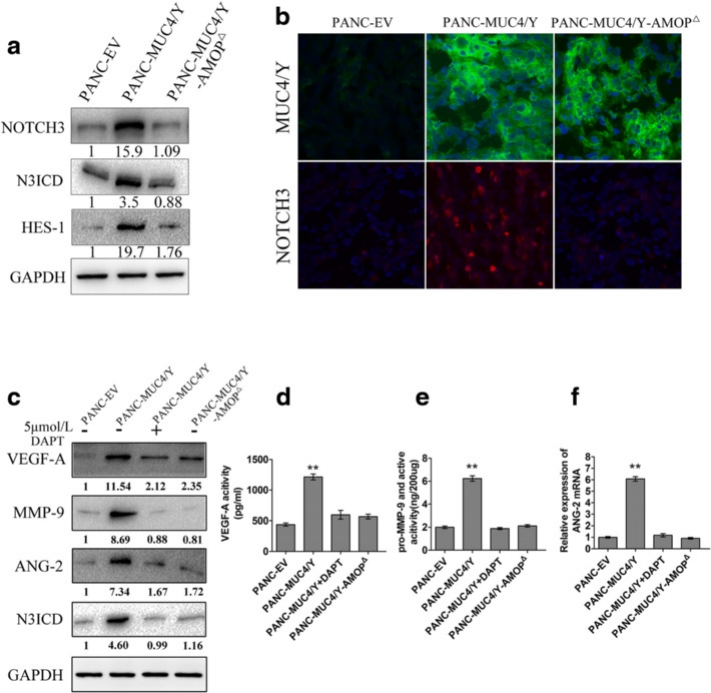
Mechanisms involved in MUC4/Y-AMOP domain regulating angiogenesis and metastasis. **a** Effects of MUC4/Y and its AMOP domain on the NOTCH3 signalling pathway (NOTCH3, N3ICD, HES-1) were assayed by western blotting. GAPDH was used as the internal control. **b** Immunofluorescence was used to assess the NOTCH3 and MUC4(MUC/Y) protein level. **c** Western blotting assays were conducted to analyse the protein level of VEGF-A, MMP-9, ANG-2 and N3ICD in different groups. GAPDH was used as the internal control. **d** The secreted level of VEGF-A in conditioned medium was detected by an ELISA kit assay. **e** The activity of MMP-9 in different groups was detected by using a Fluorokine E Human MMP-9 Activity Assay kit assay. **f** The mRNA expression of ANG-2 in different groups. DAPT: γ-secretase inhibitors (GSI) can inhibit the proteolytic processing of Notch receptors by γ-secretase, which is essential for Notch activation. **P < 0.01

To identify the pathways activated by the MUC4/Y-AMOP domain in PC, which might reveal the mechanism by which the MUC4/Y-AMOP domain enhanced tumour angiogenesis and metastasis of PC, the Real-Time qPCR and Western Blotting assays were conducted. We found that NOTCH3 mRNA (Additional file 1: Figure S3C) and protein levels (Fig. 5a) were dramatically increased in the MUC4/Y overexpressed cells. There was no obvious change of the other NOTCH receptors (NOTCH1, NOTCH2, or NOTCH4) (Additional file 1: Figure S3A, B, D). In addition, we also found that the expression of activated NOTCH3 (N3ICD), and its downstream target gene, Hes-1*,* were unregulated in MUC4/Y overexpression cells. However, the overexpression of MUC4/Y-AMOP^Δ^ did not affect the expression of NOTCH3 and its target gene, compared with the control cells (Fig. 5a, Additional file 1: Figure S3E). IHF analysis was carried out on the frozen pancreatic tumour tissue sections to confirm the expression of MUC4/Y and NOTCH3. In this assay, we found that the expression of NOTCH3 was dramatically increased in the MUC4/Y group compared with the other two groups, similar to our previous results (Fig. 5b).

### The MUC4/Y-AMOP domain has an important role in MUC4/Y, increasing the expression of VEGF-A, ANG-2 and MMP-9

It has been reported that VEGF and MMP-9 expression reduced in the stable MUC4 knockdown pancreatic cancer cell line [4, 30, 31]. The NOTCH signalling pathway may also regulate VEGF and MMP-9, which has been well documented in PC cell lines [32]. Therefore, we investigated whether VEGF-A and MMP-9 were up-regulated by MUC4/Y and the possible role of the AMOP domain in it. Western blotting was conducted to explore the expression of VEGF-A and MMP-9. We found that the protein levels of VEGF-A and MMP-9 were dramatically increased in the MUC4/Y overexpression group compared with the MUC4/Y-AMOP^Δ^ and control groups (Fig. 5c). Next, we examined the activity of VEGF-A and MMP-9. We found a marked increase in the expression of VEGF-A and MMP-9 in PANC-MUC4/Y cells than the other two groups (Fig. 5d and e).

It has been reported that the NOTCH pathway affects angiopoietin 2 (ANG-2) [33]. Therefore, we investigated whether ANG-2 expression was regulated via NOTCH3 mediated by MUC4/Y and its AMOP domain. Real-time qPCR and western blotting were used to explore the expression of ANG-2. We found that MUC4/Y could increase both mRNA and protein levels of ANG-2, whereas no change was found in the MUC4/Y-AMOP^Δ^ group (Fig. 5c and f).

To confirm that the MUC4/Y-AMOP domain up-regulates the expression of VEGF-A, MMP-9, and ANG-2 via NOTCH3 signalling, we added an experimental group as following: PANC-MUC4/Y + DAPT (5umol/L, 48 h; γ-secretase inhibitors (GSI) can inhibit the proteolytic processing of NOTCH receptors by γ-secretase, which is essential for NOTCH activation) [34]. We found that DAPT could counteract the effects: the increased expression or activities of VEGF-A, MMP-9, and ANG-2 (Fig. 5c-f), which could lead to angiogenesis and metastasis of PC cells.

## Discussion

The MUC4 protein is a large transmembrane type I glycoprotein that contains several important functional domains. However, because of the technical restrictions in cloning and modifying the large cDNA of MUC4, direct experimental proof for the role of individual MUC4 extracellular domain in cancer development and progression has been difficult to obtain.

MUC4/Y, a transcript variant of MUC4, lacks the transcription from exon 2 (the tandem repeat domains) compared with the full length MUC4 [16]. Thus, the validated functions of this transcript variant may help us to understand the potential functions of MUC4 in PC development and progression. Because MUC4/Y lacks the large fragment of exon 2, we can study the functions of MUC4/Y and the domains it contains (NIDO, AMOP, vWD, EGF-like) using the overexpression lentivirus system.

Some previous studies showed that MUC4 and HER2 interact physically and transduce intracellular signals to promote a series of biological processes in PC cells [34, 35]. In this study, we conducted the western blot assay again to check the expression of total-ErbB2/phosphor-ErbB2. We found that the expression of total-ErbB2 and phosphor-ErbB2 were increased in PANC-MUC4/Y group, while no obvious change of them was found when the AMOP domain was deleted. So, we concluded that MUC4/Y might activate the ErbB2, while not through the AMOP domain (Additional file 1: Figure S3F).

Other laboratories reported that the MUC4-NIDO domain could contribute to the MUC4-mediated metastasis of PC cells by expressing the engineered MUC4 (mini MUC4) and MUC4 without the NIDO-domains. In these studies, they showed that MUC4-NIDO domain-mediated metastasis might be partly due to its interaction with the endogenous fibulin-2 protein [36]. Komatsu et al. showed that overexpression of Muc4 (rat Muc4) or MUC4 hindered integrin-mediated cell-cell and cell-ECM adhesion in vitro because of the large size of MUC4 [37, 38]. In order to avoid changing the spatial structure, we focused on MUC4/Y, a natural transcript variant of MUC4, lacks fragment from exon 2 (~120 KD), from another point of view: signal transduction, in the current study. The results of real-time qPCR and western blotting experiments showed that MUC4/Y could activate NOTCH3 signalling, and the loss of the MUC4/Y-AMOP domain decreased the activity. The NOTCH signalling is abnormally activated in many human malignancies, including pancreatic cancer [39–43]. It is known to play critical roles in the processes of tumour cell angiogenesis, proliferation, invasion, and metastasis [32, 40, 44–47]. Therefore, MUC4/Y-AMOP domain-promoted tumour angiogenesis and metastasis may be partly due to the activation of NOTCH3.

Many studies have documented that VEGF is a critical mediator of angiogenesis and metastasis [48–52]. Here, we showed that MUC4/Y-overexpression increased VEGF-A protein expression. We also found a marked increase in the secreted form of VEGF-A in the conditioned medium of MUC4/Y-overexpressed cells. However, the loss of the AMOP domain reduced the expression of VEGF-A induced by MUC4/Y.

Another important class of molecules involved in tumour metastasis and angiogenesis are the MMPs. It is known that MMPs are critically involved in the processes of tumour cell invasion and metastasis and that MMP-9 is directly associated with angiogenesis and metastatic processes [4, 32, 46, 53, 54]. In this study, we showed that overexpression of MUC4/Y increased MMP-9 protein levels and activity.

Apart from VEGF and MMPs, ANG-2 plays crucial role in tumour angiogenesis [55–58]. Although the role and mechanism of ANG-2 in tumour angiogenesis has not been fully clarified, experimental studies have demonstrated a close relationship of VEGF and ANG-2 functions in angiogenesis. ANG-2 promotes vessel sprouting in the presence of abundant VEGF, whereas ANG-2 contributed to vessel regression in the absence of VEGF [59, 60]. There are no reports of a connection between ANG-2 and MUC4/Y (MUC4) yet. However, it has been reported that ANG-2 is involved in NOTCH signalling. In this study, we found that MUC4/Y could increase both mRNA and protein levels of ANG-2, whereas no change was found in the MUC4/Y-AMOP^Δ^ group.

Finally, we found that DAPT could counteract the increased expression or activities of Hes-1, VEGF-A, MMP-9, and ANG-2 caused by MUC4/Y overexpression. This result demonstrated that NOTCH3 signalling was the key mechanism underlying these complex phenomena.

Based on our results, we speculate one possible mechanism that the MUC4/Y-AMOP domain induces invasion and angiogenesis by activating NOTCH3 to up-regulate the downstream functional genes, such as VEGF-A, MMP-9, and ANG-2. In addition, taking the membrane location into account, we presume that the MUC4/Y-AMOP domain may be the key region involved in MUC4/Y binding to NOTCH3. This mechanism may be important in various biological processes mediated by MUC4. In subsequent experiments, we will attempt to validate our ideas and further ascertain the precise molecular interaction between the MUC4/Y-AMOP domain and NOTCH3. When the functions of MUC4 (MUC4/Y) and AMOP domain in pancreatic cancer are clarified, we could design some specific sequence such as aptamer/siRNA to silence them, in order to reduce, or even block the malignant biological behavior of pancreatic cancer.

Furthermore, we are the first to use a transcript variant model to study the specific domains of a high molecular weight membrane protein that is difficult to overexpress. In the future, the method we used to investigate the AMOP domain can be a model for studying other high molecular weight membrane proteins. The data in this study are also consistent with the hypothesis that MUC4 is a multifunctional target for the treatment of PC.

## Conclusions

In conclusion, the present study provides evidence that AMOP domain plays the key role in MUC4/Y(MUC4)-mediated tumour angiogenesis and metastasis of PC cells partly through the NOTCH3 signalling and its downstream target genes: VEGF-A, MMP-9 and ANG-2. This mechanism could be the potential therapeutic target of PC. Furthermore, the method we used to investigate the AMOP domain could be a model for studying other high molecular weight membrane proteins.

## Abbreviations

HUVEC, is short for human umbilical venous endothelial cells; PC, is short for human pancreatic cancer; qRT-PCR, is short for quantitative reverse transcription polymerase chain reaction

## Additional file

Additional file 1: The attached table and figures. (PDF 549 kb)
