# Erratum to: The role of the AMOP domain in MUC4/Y-promoted tumour angiogenesis and metastasis in pancreatic cancer

**DOI:** 10.1186/s13046-017-0505-5

**Published:** 2017-02-22

**Authors:** Jie Tang, Yi Zhu, Kunling Xie, Xiaoyu Zhang, Xiaofei Zhi, Weizhi Wang, Zheng Li, Qun Zhang, Linjun Wang, Jiwei Wang, Zekuan Xu

**Affiliations:** 1 Department of General Surgery, the First Affiliated Hospital of Nanjing Medical University, Nanjing, Jiangsu China; 2 Department of Pediatric Surgery, Nanjing Children’s Hospital Affiliated to Nanjing Medical University, Nanjing, Jiangsu China; 3 Department of General Surgery, the People’s Hospital of Bozhou, Bozhou, Anhui China; 4 Department of General Surgery, Huai’an People’s Hospital, Xuzhou Medical College, Huai’an, Jiangsu China; 5 Department of General Surgery, Affiliated Hospital of Nantong University, Nantong, Jiangsu China

## Erratum

In the original publication of this article [1], the funding number of the NSFC-NIH project was listed incorrectly. This should have been 81361120398, and not 812111519, which belongs to the researching project of another group.

Please see amended acknowledgements section below:

This work was partially supported by the National Natural Science Foundation Project of International Cooperation (NSFC-NIH, 81361120398), the National Natural Science Foundation of China (81272712, 30901421), The Program for Development of Innovative Research Team in the First Affiliated Hospital of NJMU, the Priority Academic Program Development of Jiangsu Higher Education Institutions (PAPD, JX10231801), and the translational research of early diagnosis and comprehensive treatment in pancreatic cancer (The research Special Fund For public welfare industry of health, 201202007).
