# Supplementary material for: The role of the AMOP domain in MUC4/Y-promoted tumour angiogenesis and metastasis in pancreatic cancer
Source: J Exp Clin Cancer Res. 2016 Jun 10;35:91. doi: 10.1186/s13046-016-0369-0 (PMC4902942; doi:10.1186/s13046-016-0369-0)
Supplement: Additional file 1: — The attached table and figures. (PDF 549 kb) [file 13046_2016_369_MOESM1_ESM.pdf]

The role of the AMOP domain in MUC4/Y-promoted tumour angiogenesis and metastasis in pancreatic cancer

Jie Tang, Yi Zhu, Kunling Xie, Xiaoyu Zhang, Xiaofei Zhi, Weizhi Wang, Zheng Li, Qun Zhang, Linjun Wang, Jiwei Wang, Zekuan Xu

Supplemental table 1: the "aa" positions of the MUC/Y、the AMOP domain、and the MUC4/Y-AMOP△.

**name**	**sequence**
MUC4/Y (3513bp)	ATGAAGGGGGCACGCTGGAG......CAGCTGAGGCC TTGCCTTGA
AMOP domain(949−1296bp, 347bp)	GAAAGGCCCAACTACCGTCT......CCCTGTACCAG CAGAGGCGG
MUC4/Y-AMOP△(3183bp)	ATGAAGGGGGCACGCTGGAG......CAGCTGAGGCC TTGCCTTGA

Supplemental Fig.1

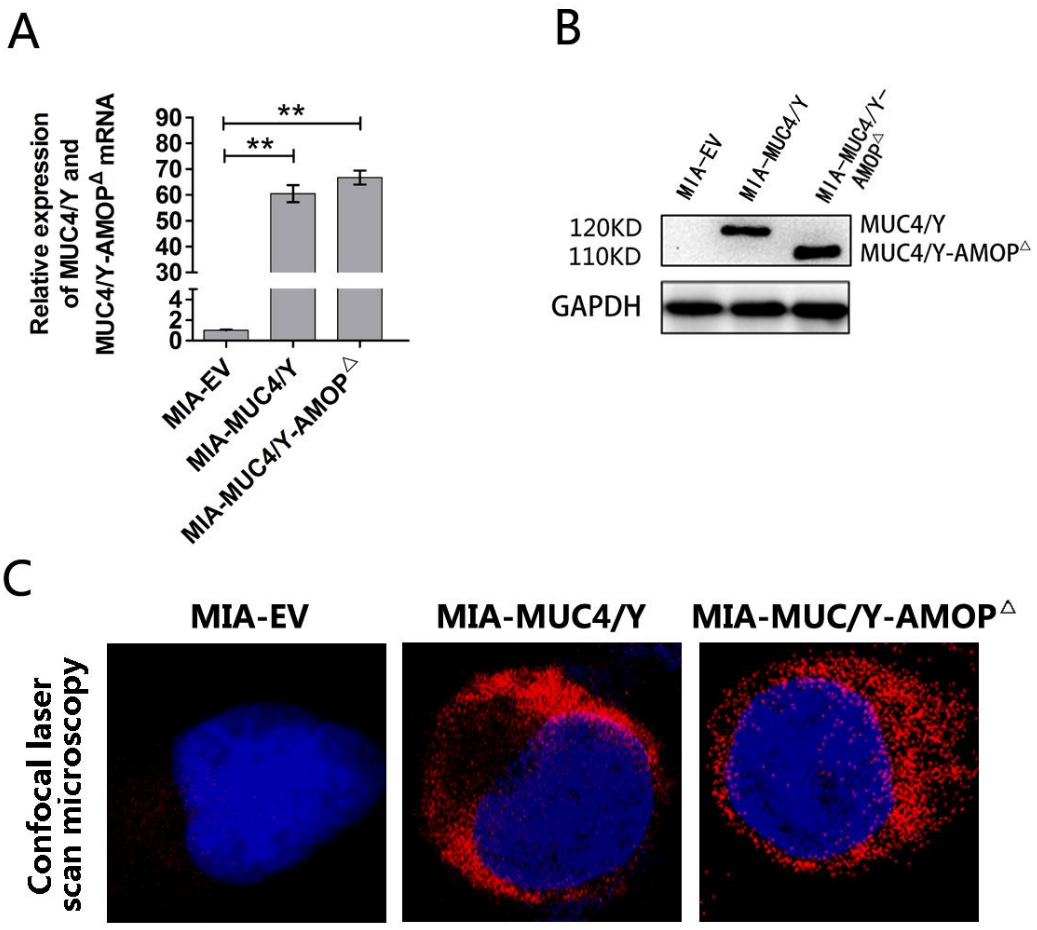

Figure Legends: MUC4/Y and MUC4/Y-AMOP domain-deletion constructs and their stable expression in MIApaca-2 (MIA) cells. (a) MUC4/Y expression was analysed by real-time PCR in different MIA derived sub-lines. GAPDH was used as an internal control. (b) Western blotting was conducted to analyse the protein level of MUC4/Y in different groups. GAPDH was probed as an internal control. (c) Cell confocal immunofluorescence assay showed that the MUC4/Y and the truncated protein (MUC4/Y-AMOP$^{\Delta}$) could anchor on the cytomembrane and cytoplasm.**P<0.01.

Supplemental Fig.2

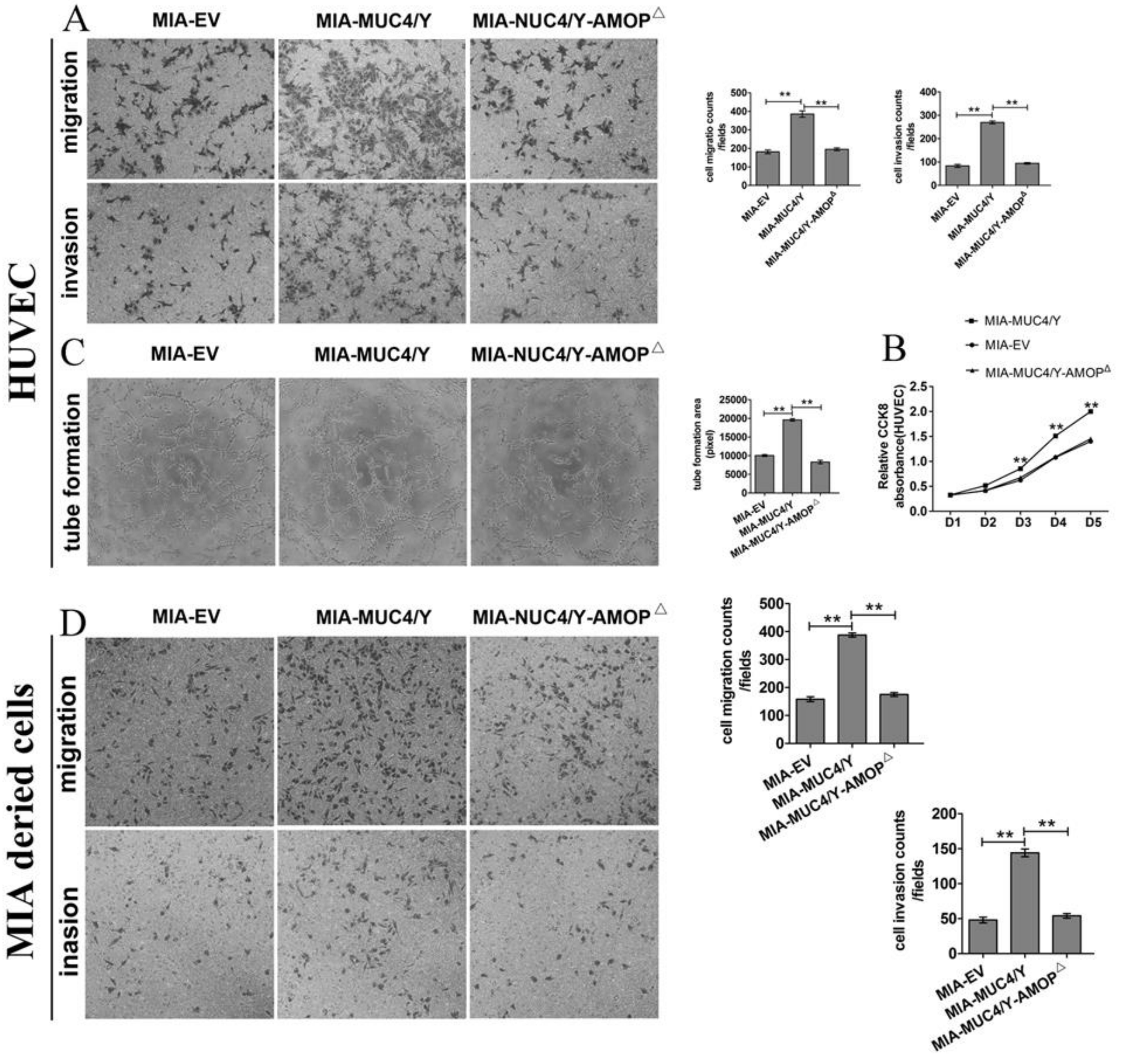

Figure legends: Effects of the MUC4/Y-AMOP domain-deletion on the migration and invasion of Miapaca-2 derived sub-lines and the conditioned medium on the tube formation, proliferation, migration, and invasion of HUVEC. (a) Trans-well migration and Matrigel invasion assays of HUVEC were performed in the conditioned medium of different groups. (b) CCK8 assay. The OD value was used to assess the proliferation of HUVEC in the conditioned medium of different groups. (c) Tube formation assays of HUVEC. The area of network was calculated with Image Pro Plus

6.0. (d) Trans-well migration and Matrigel invasion assays of Miapaca-2 derived sub-lines. The number of cells that migrate or invade to the lower surface of the inserts chamber were stained and photographed. *P<0.05, **P<0.01.

Supplemental Fig.3

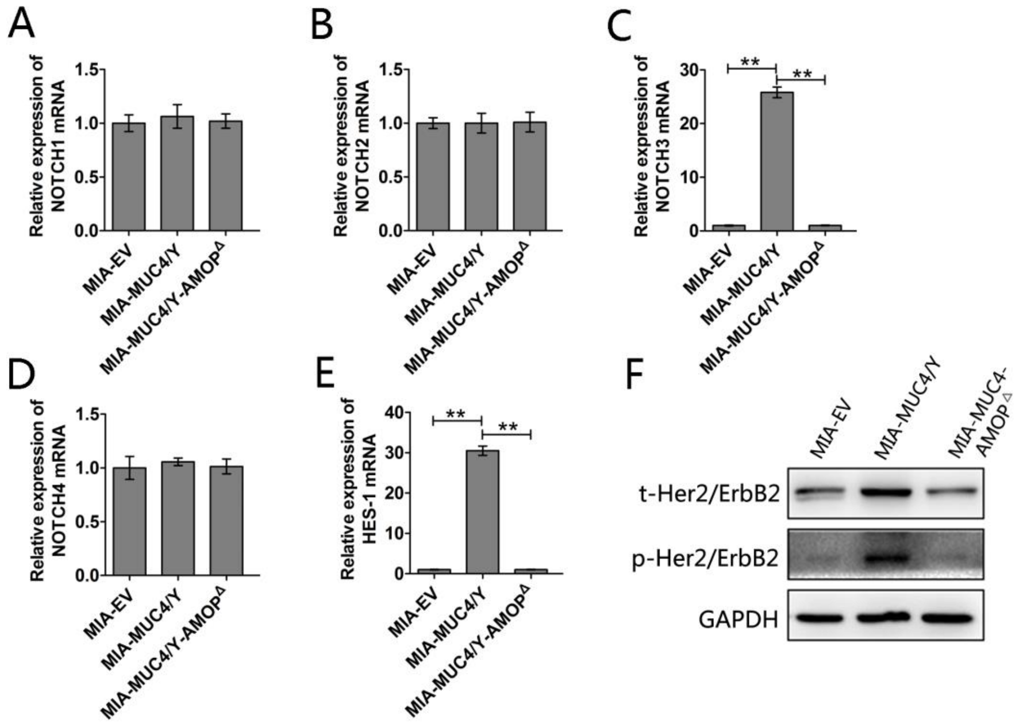

Figure Legends: The relative expresssion of NOTCH1, NOTCH2, NOTCH3, NOTCH4 receptors, HES-1 and Her2/ErbB2. QRT-PCR and Western Blotting were used to analyze the expression levels of them. (a) The relative expression of NOTCH1 mRNA. (b) The relative expression of NOTCH2 mRNA. (c) The relative expression of NOTCH3 mRNA. (d) The relative expression of NOTCH4 mRNA. (e) The relative expression of HES-1 mRNA. (f) The protein expression of total-Her2/ErbB2 (t-Her2/ErbB2) and phosphorylation-Her2/ErbB2 (p-Her2/ErbB2).
